# An effective assessment method of spinal flexibility to predict the initial in-orthosis correction on the patients with adolescent idiopathic scoliosis (AIS)

**DOI:** 10.1371/journal.pone.0190141

**Published:** 2017-12-21

**Authors:** Chen He, Michael Kai-Tsun To, Jason Pui-Yin Cheung, Kenneth Man-Chee Cheung, Chi-Kwan Chan, Wei-Wei Jiang, Guang-Quan Zhou, Kelly Ka-Lee Lai, Yong-Ping Zheng, Man-Sang Wong

**Affiliations:** 1 Interdisciplinary Division of Biomedical Engineering, The Hong Kong Polytechnic University, Hung Hom, Kowloon, Hong Kong; 2 Department of Orthopaedics and Traumatology, The University of Hong Kong, Pokfulam, Hong Kong; 3 Department of Prosthetics and Orthotics, The Duchess of Kent Children's Hospital, Sandy Bay, Hong Kong; 4 College of Computer Science and Technology, Xihu District, Hangzhou, Zhejiang Province, China; 5 State Key laboratory of Biological Science and Medical Engineering, Southeast University, Xuanwu District, Nanjing, Jiangsu Province, China; Tokai University, JAPAN

## Abstract

**Background:**

Spinal flexibility is an essential parameter for clinical decision making on the patients with adolescent idiopathic scoliosis (AIS). Various methods are proposed to assess spinal flexibility, but which assessment method is more effective to predict the effect of orthotic treatment is unclear.

**Objective:**

To investigate an effective assessment method of spinal flexibility to predict the initial in-orthosis correction, among the supine, prone, sitting with lateral bending and prone with lateral bending positions.

**Methods:**

Thirty-five patients with AIS (mean Cobb angle: 28° ± 7°; mean age: 12 ± 2 years; Risser sign: 0–2) were recruited. Before orthosis fitting, spinal flexibility was assessed by an ultrasound system in 4 positions (apart from standing) including supine, prone, sitting with lateral bending and prone with lateral bending. After orthosis fitting, the initial in-orthosis correction was routinely assessed by whole spine standing radiograph. Comparisons and correlation analyses were performed between the spinal flexibility in the 4 positions and the initial in-orthosis correction.

**Results:**

The mean in-orthosis correction was 41% while the mean curve correction (spinal flexibility) in the 4 studied positions were 40% (supine), 42% (prone), 127% (prone with lateral bending) and 143% (sitting with lateral bending). The correlation coefficients between initial in-orthosis correction and curve correction (spinal flexibility) in the 4 studied positions were r = 0.66 (supine), r = 0.75 (prone), r = 0.03 (prone with lateral bending) and r = 0.04 (sitting with lateral bending).

**Conclusions:**

The spinal flexibility in the prone position is the closest to and most correlated with the initial in-orthosis correction among the 4 studied positions. Thus, the prone position could be an effective method to predict the initial effect of orthotic treatment on the patients with AIS.

## Introduction

Adolescent idiopathic scoliosis (AIS) is a complex three-dimensional (3D) deformity of the spine and rib cage, which occurs predominantly in pre-pubertal girls [1]. The prevalence of scoliosis is 2–4%, which is diagnosed by a Cobb angle greater than 10° [2]. Currently, options for AIS treatment include: observation, for the patients with small curves or at skeletal maturity; orthotic treatment, for those with moderate curves and growth potential; and surgery, for those with severe curves [3]. Until now, orthotic treatment has served as an important non-surgical treatment for the patients with moderate AIS [4].

The response of the scoliotic spine to the initial orthosis application (initial in-orthosis correction) is essential to determine the long-term treatment effectiveness [5]. In the current practice, some clinicians estimate the in-orthosis effectiveness by clinical experience and use it to assist orthosis design. This empirical practice makes treatment planning less scientific and evidence-based, which would consequently affect the treatment effectiveness. Or some clinicians aim to achieve 40–50% correction of the initial curvature [6–8] and use this general standard to guide orthosis design. However, the initial in-orthosis corrections are usually different among patients due to individualized spinal conditions, using a general standard for all patients makes the tailor-made orthosis less personalized and patient-specific. To optimize the current practice, quantitative prediction of initial in-orthosis correction according to individual patient’s condition is necessary.

Spinal flexibility has been used to predict the initial in-orthosis effectiveness since more flexible spines are estimated with better correction by orthosis. Some used the curve correction revealed in supine position (supine flexibility) to predict the curve correction obtained by spinal orthosis [9, 10], while detailed statistical results were not provided in these studies. Kuroki et al. proposed standing with traction position to assess spinal flexibility and predict orthotic correction [11], but its correlation with the in-orthosis correction was diverse due to varying maturity status of their patients (at 9–18 years with Risser 0–5). A recent study reported that the curve angle in the supine with lateral bending position was the same as the initial in-orthosis correction with a mean difference of 0.28° [12]. However, this finding may only be applicable to the Providence nighttime orthosis which was used in their study. Other methods, such as supine with traction and fulcrum bending, predict surgical correction [13, 14] but may not predict the orthotic correction. At present, the method of spinal flexibility assessment that offers an effective prediction of the initial in-orthosis correction is still unknown, a comparison among these methods is deserved. However, this comparison may not be feasible in the past because it requires X-ray taking at different body positions that exposes the patients to more radiation. An ultrasound technique would be an option for the radiation-free comparison since 3-D ultrasound (US) has been proved to be a reliable and valid technique to assess scoliosis [15–17]. Therefore, this study aims to investigate an effective assessment method of spinal flexibility to predict the initial in-orthosis correction using US technique.

## Materials and methods

A prospective cohort study was conducted at a tertiary referral scoliosis clinic. The inclusion criteria consisted of the patients with AIS at (1) Cobb angle: 25°- 45° in major curve; (2) age: 10–16 years; (3) Risser sign: 2 or less; (4) prior to the first orthotic treatment. A sample size of 28 subjects was calculated (assuming that effect size (d) = 0.5; statistical power (1-β) = 0.8; level of significance (α) = 0.05 used for 2-tailed T-test). The Human Subjects Ethics Sub-Committee of The Hong Kong Polytechnic University approved this research. Written consents were obtained from all the subjects and their guardians. The individual in the figure has given written informed consent (as outlined in PLOS consent form) to publish the details.

**Fig 1:**
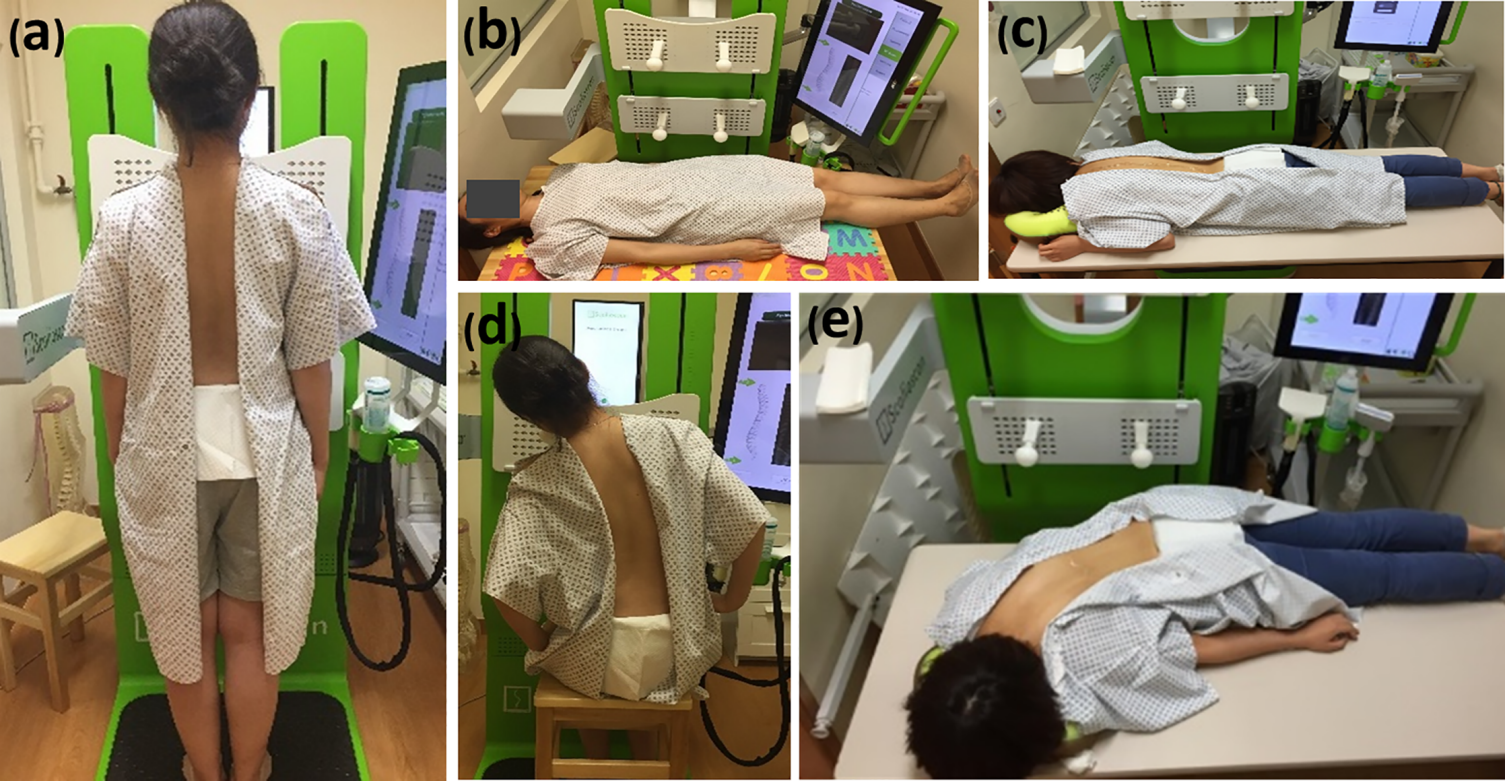
Positions for spinal flexibility assessment. (a) standing (b) supine (c) prone (d) sitting with lateral bending (e) prone with lateral bending.

Before orthosis fitting, spinal flexibility was assessed by an ultrasound system “Scolioscan” (Model SCN801, Telefield Medical Imaging Ltd, Hong Kong) in standing and other 4 positions, including supine, prone, sitting with lateral bending and prone with lateral bending (Fig 1) in a random order via drawing lots. In the standing position, patients were instructed to look forward and keep body straight with pelvis level and feet shoulder-width apart. In the recumbent positions (supine / prone), patients were instructed to lie down facing up / down on a scanning couch, trunk and legs straight, and arms beside trunk. In the lateral bending positions (sitting / prone with lateral bending), patients were instructed to bend towards the convexity of the scoliosis from neutral to the maximum limit (bending to both sides if double curves), and hold for 30 seconds for ultrasound scanning. A laser alignment device was used to monitor the alignment of the pelvis during bending assessment. All subjects were requested to practice the positions 3 times to meet the above-mentioned requirements before ultrasound assessment. Ultrasound scanning was repeated for two times with one-minute rest in between in each position. A team of orthotists with more than 5-year experience applied standardized protocol to design and fabricate the symmetric underarm rigid spinal orthoses for subjects. All orthotists were blind to the ultrasound flexibility measurements. After the adaption period of orthosis wearing (2–3 weeks), the initial in-orthosis correction was captured by a whole spine postero-anterior standing radiograph when the orthosis was fitted for more than two hours for achieving the maximum in-orthosis correction [18]. The finalized in-orthosis X-ray result was regarded as the initial in-orthosis correction, if major modification of the orthosis was made and in-orthosis X-ray was retaken.

The ultrasound images were measured using a standardized method as stated in Zheng et al.’s study [15]. The spinous process of each vertebra was marked, and the levels of the upper and lower end-vertebrae were selected according to the standing radiograph, then, a line was drawn to join the spinous process at each level, and the curve angle basing on the selected end-vertebrae was calculated automatically by a purpose-designed software.

Statistical analyses were performed using the IBM SPSS Statistics, Version 21 (IBM, Armonk, New York, USA). The curve angle in each position was the average result of the two scans. The curves were divided into subgroups as mild thoracic curves (<25°), moderate thoracic curves (25°~45°), mild lumbar curves (<25°) and moderate lumbar curves (25°~45°) for analyses, in order to reduce the influence of curve magnitude and location on the corresponding correlation between spinal flexibility and in-orthosis correction. The spinal flexibility and initial in-orthosis correction were computed as: Spinal flexibility = (Angle _US standing_−Angle _US in given position_) / Angle _US standing_; Initial in-orthosis correction = (Angle _X-ray standing_−Angle _X-ray in-orthosis_) / Angle _X-ray standing_. The confidence interval was set at 95% (p<0.05). One-way repeated analysis of variance (ANOVA) with least-squared differences (LSD) post-hoc tests were performed to compare the 4 spinal flexibilities and initial in-orthosis correction. The Pearson product-moment correlation was used to determine the correlation between the 4 spinal flexibilities and the in-orthosis correction, with correlation coefficient 0.00–0.24 indicating no correlation, 0.25 to 0.49 indicating low correlation, 0.50–0.74 indicating moderate correlation, and 0.75–1.00 indicating good correlation [19].

## Results

**Fig 2:**
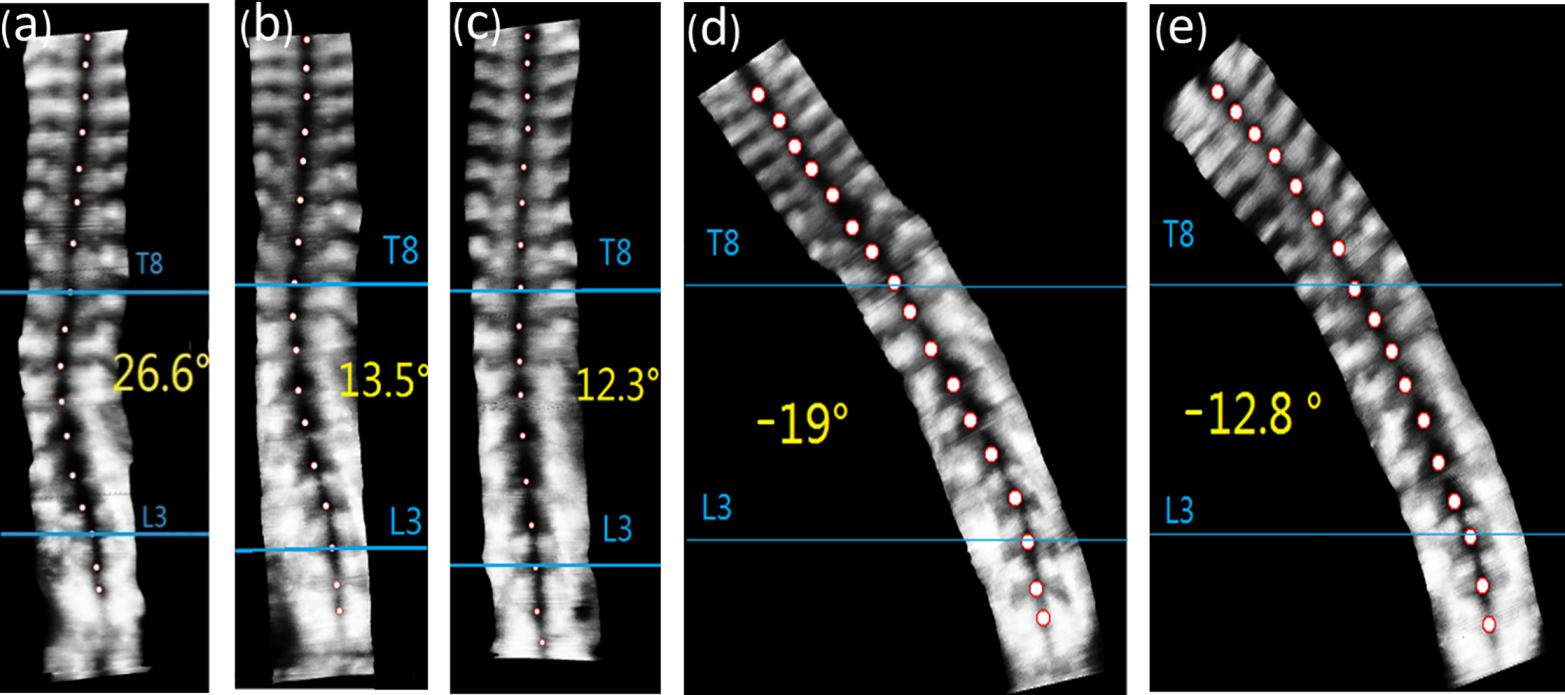
Ultrasound images in (a) standing position (b) supine position (c) prone position (d) sitting with lateral bending position (e) prone with lateral bending position. The left thoracolumbar curve ranged from T8 to L3 (apex at T11) with the magnitude of 26.6° in standing position, 13.5° in supine position, 12.3° in prone position, -19° in sitting with lateral bending position, and -12.8° in prone with lateral bending position (negative value refers to the curve being corrected to the opposite direction).

Totally 35 subjects (mean Cobb angle: 28° ± 7°; mean age: 12 ± 2 years; Risser sign: 0–2) were recruited. The patient demographic data are presented in Table 1. The spinal flexibility assessed in the 4 positions and the initial in-orthosis correction of each group is shown in Table 2. The typical ultrasound images of a patient are shown in Fig 2.

**Table 1:** Patient demographic data.

Number of patients	Sex	Age	Risser sign	BMI	Cobb angle	Curve pattern
n = 35 (67 curves)	32 females 3 males	12±2 years	0–2	19 ± 2 kg/m^2^	28° ± 7° (15° - 45°)	double curve (n = 32) and single curve (n = 3)

**Table 2:** Spinal flexibility and in-orthosis correction.

Group	Pre-orthosis Standing (X-ray*)	Spinal Flexibility (US*)				In-orthosis Correction (X-ray*)
		Supine	Prone	Sitting bending	Prone bending	
**Mild thoracic curves (n = 10)**	22° ± 2°	41% ± 22% ^a^^,^^b^	43% ± 18% ^a^^,^^b^	159% ± 46%	135% ± 20%	38% ± 21%
**Moderate thoracic curves (n = 24)**	32° ± 5°	36% ± 19% ^a^	37% ± 20% ^a^	126% ± 39%	116% ± 35%	33% ± 19%
**Mild thoracolumbar/lumbar curves (n = 13)**	20° ± 4°	46% ± 23% ^a^	45% ± 14% ^a^^,^^b^	174% ± 66%	149% ± 34%	48% ± 24%
**Moderate thoracolumbar/lumbar curves (n = 20)**	31° ± 5°	42% ± 16% ^a^	46% ± 17% ^a^^,^^b^	137% ± 64%	121% ± 35%	48% ± 24%
**Overall curves(n = 67)**	28° ± 7°	40% ± 19% ^a^	42% ± 18% ^a^^,^^b^	143% ± 56%	127% ± 34%	41% ± 23%

* X-ray: X-ray assessment, US: ultrasound assessment.

^a^ no significant difference with the corresponding in-orthosis correction (p>0.05).

^b^ good correlation with the corresponding in-orthosis correction (r>0.75).

**Fig 3:**
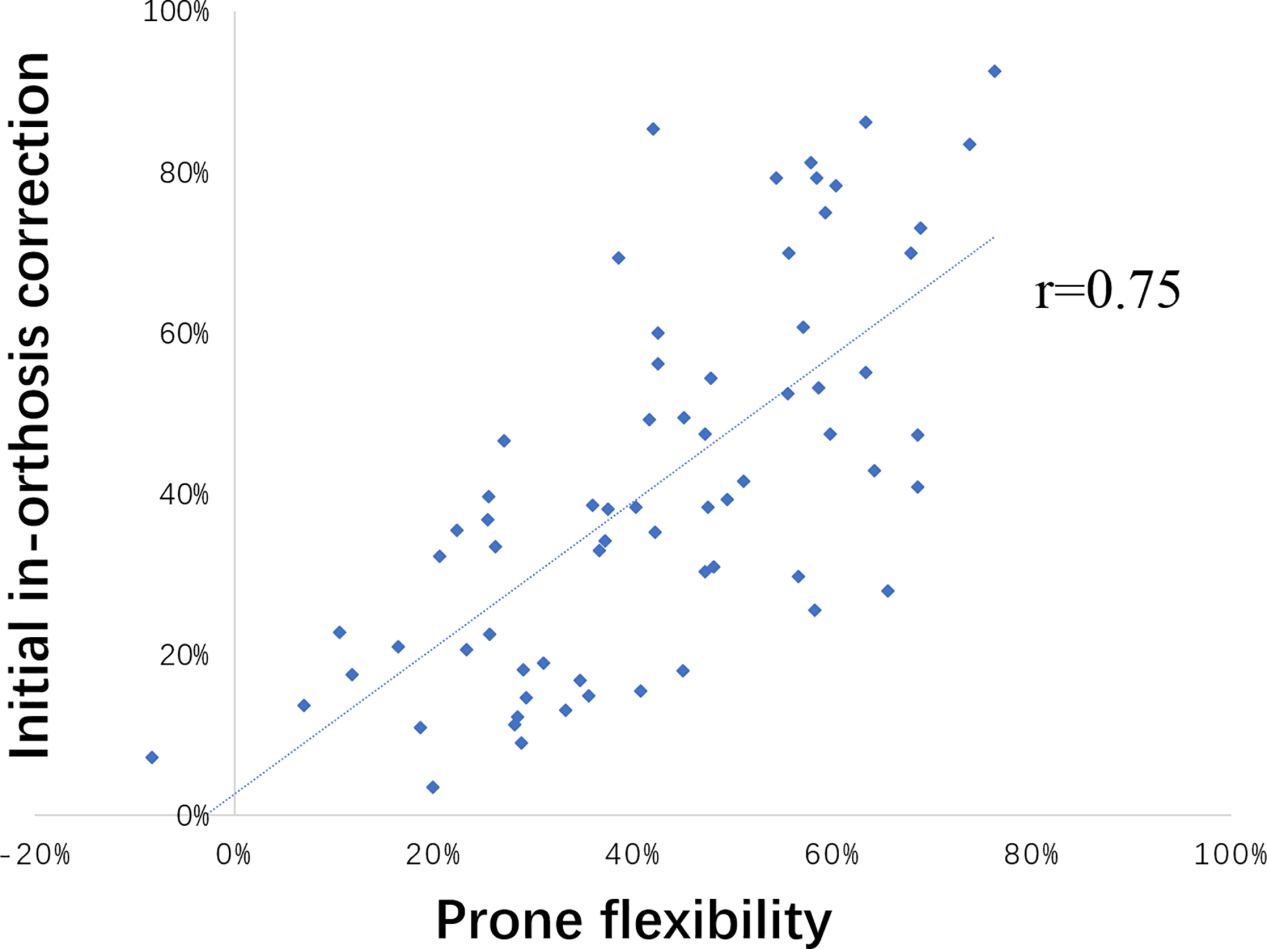
Correlation between prone flexibility and initial in-orthosis correction.

For the recumbent flexibilities in the overall curves, no significant differences were observed between the supine / prone flexibility and in-orthosis correction (p = 0.67 and 0.59 respectively). The supine flexibility showed moderate correlation while the prone flexibility showed good correlation with the in-orthosis correction (r = 0.66 and 0.75 respectively) (Fig 3). In the subgroups, no significant differences were observed between the prone / supine flexibility and in-orthosis correction (p>0.05). The supine flexibility was moderately correlated with the in-orthosis correction in all the subgroups (0.5<r<0.75) except the mild thoracic curves with good correlation (r = 0.82). The prone flexibility showed good correlation with the in-orthosis correction in all the subgroups (r>0.75) except moderate correlation (r = 0.67) in the moderate thoracic curves.

For the lateral bending flexibilities in the overall curves, both the sitting and prone with lateral bending demonstrated higher correction (143% and 127% respectively) than the in-orthosis correction (41%), and did not correlate with the in-orthosis correction (r = 0.04 and 0.03 respectively). In the subgroups, no correlation between the lateral bending flexibilities and in-orthosis correction were also found.

The standing curve angle assessed by ultrasound was significantly lower than that assessed by X-ray (p<0.05), they were good correlated both in overall curves (r = 0.77) and subgroups (r>0.75).

## Discussion

This novel study utilized ultrasound to assess the spine flexibility in 4 positions, and found that the spinal flexibility in the prone position is the closest to and most correlated with the initial in-orthosis correction. This finding may indicate that the spinal flexibility in the prone position provides an effective method to predict the initial effect of orthotic treatment in the patients with AIS.

The spinal flexibility (curve correction) in recumbent positions were found of no significant difference to the in-orthosis correction in this study. This is in accordance with the previous study which reported that supine curve angle before orthotic treatment was close to the curve angle after fitting orthosis [10]. In an erect position, gravitational effect adds axial loading on the spine while muscles maintain a balanced trunk alignment. In a recumbent position, the gravitational effect on the spine is eliminated axially and some muscle groups relax, meanwhile the supporting surface exerts an upward force to the spine. Therefore, a lying down position can reduce the spinal curvature and demonstrate recumbent flexibility. Spinal orthosis corrects the spinal curvature mainly via the correction pads that apply posterolateral forces to the spine [20]. The correction effect by the two mechanisms (lying down and applying orthosis) were found similar, which may suggest that the curve correction (spinal flexibility) in recumbent positions can be used to predict the orthotic correction prior to orthosis fitting.

Even though recumbent flexibility (both supine and prone flexibility) are not significantly different from the initial in-orthosis correction, the prone flexibility is more predictive to the in-orthosis correction because the prone flexibility showed a good correlation (R = 0.75) but the supine flexibility showed only moderate correlation (R = 0.66) with the in-orthosis correction. Therefore, a prone position can be an alternative to the supine position for spinal flexibility assessment. Integrating prediction of in-orthosis correction by prone flexibility assessment at the pre-orthosis stage can assist clinicians to differentiate the patients who are unlikely to benefit from orthotic treatment (such as a patient with an expectation of less than 20% in-orthosis correction [21]) thus preventing unnecessary orthosis application. In addition, individualized orthosis design according to the prone flexibility rather than personal experience or general standard makes the orthosis planning process more evidence-based and patient-specific. As the prone position with manual correction or prone position under general anesthesia were reported to have potential predictability to the postoperative correction [22, 23], future studies are deserved to explore the feasibility of using prone flexibility to assist surgical planning.

The lateral bending flexibility (both sitting and prone with lateral bending) were found beyond 100% and showed no correlation with the orthotic correction in this study, while the previous studies reported lateral bending flexibility to be 40–80% [14, 24–26] and correlated with the surgical correction [27, 28]. The disagreement may be due to the difference of studied patients and treatment methods: patients with moderate scoliosis versus patients with severe scoliosis (prediction of orthotic correction versus prediction of surgical correction). Clin et al. found that the bending moments at the curve apex were correlated to the in-orthosis correction via finite element analysis of three scoliotic models, while a direct assessment of bending moment at apex was less feasible on human subject trials. Thus, the other clinical parameter, curve angle in lateral bending position and corresponding bending flexibility, were analyzed in this study [29]. No correlation between lateral bending flexibilities and initial orthotic correction found in this study may indicate that the maximum flexibility revealed by lateral bending is far beyond the curve correction by the symmetric underarm spinal orthosis. Some orthoses with asymmetric design aims to take up the maximum flexibility of the spine and keep the curvature at the maximum correction position (such as Charleston Bending Orthosis), whether the lateral bending flexibility can predict the treatment effect of these orthoses needs further studies.

The influence of curve magnitude and location on the correlation between spinal flexibility and in-orthosis correction was not obvious in this study. The correlation between recumbent flexibility and in-orthosis correction was similar in mild and moderate curves (r = 0.6~0.8 and 0.6~0.9 respectively), and similar in thoracic and thoracolumbar/lumbar curves (r = 0.6~0.8 and 0.6~0.9 respectively) as well. No correlation (r<0.25) was found between the lateral bending flexibility and in-orthosis correction regardless of mild or moderate curves, thoracic or thoracolumbar/lumbar curves. These findings indicated that the correlation between spinal flexibility and in-orthosis correction demonstrated a consistent trend in the subgroups and overall curves.

Ultrasound technique was firstly used in this study to assess the spinal flexibility on the patients with AIS and a high feasibility was found. The reliability and validity of using ultrasound to assess scoliosis has been well established previously [15–17], which agrees with this study: a good correlation between the ultrasound and radiographic assessment (r = 0.77). It is also found that the curve angle assessed by ultrasound was lower than that assessed by X-ray. The possible reason is that the vertebra posterior elements (e.g. spinous process) rather than the endplates of vertebral bodies were identified as landmarks for curve angle calculation in ultrasound images [15], which tends to reveal smaller curve angle than Cobb angle in X-ray images [30]. Spinal flexibility is essential for orthosis treatment planning but not always be quantitatively assessed due to extra radiation exposure. Ultrasound allows radiation-free assessment of spinal flexibility in an efficient and cost-effective way. Therefore, ultrasound technique can potentially supplement radiography to assess the spinal flexibility and further optimize the orthosis planning process.

This study has some limitations. The spinal flexibility on the coronal plane was discussed while the comprehensive 3-D information of the spine will be analyzed after the full 3D image analysis software is ready. Besides, the initial in-orthosis correction was only assessed by radiograph because the US scanning transducer (5 cm in width) is not applicable to the current orthosis design (2 cm posterior opening in width). A smaller transducer will be used in the future study.

## Conclusion

The spinal flexibility in the prone position is the closest to and most correlated with the initial in-orthosis correction among the 4 studied positions. Thus, the prone position could be an effective method to predict the initial effect of orthotic treatment on the patients with AIS.

## Supporting information

S1 Table Spinal flexibility of ultrasound assessments and initial in-orthosis correction of X-ray assessments. (XLSX)
